# Participant Profiles According to Recruitment Source in a Large Web-Based Prospective Study: Experience From the Nutrinet-Santé Study

**DOI:** 10.2196/jmir.2488

**Published:** 2013-09-13

**Authors:** Emmanuelle Kesse-Guyot, Valentina Andreeva, Katia Castetbon, Michel Vernay, Mathilde Touvier, Caroline Méjean, Chantal Julia, Pilar Galan, Serge Hercberg

**Affiliations:** ^1^Université Paris 13, Sorbonne Paris Cité, UREN, Inserm (U557); Inra (U1125), CnamBobignyFrance; ^2^Université Paris 13, Sorbonne Paris Cité, USEN, Institut de Veille SanitaireBobignyFrance; ^3^Département de Santé PubliqueHôpital Avicenne (AP-HP)BobignyFrance

**Keywords:** cohort study, Internet, selection bias, population characteristics

## Abstract

**Background:**

Interest in Internet-based epidemiologic research is growing given the logistic and cost advantages. Cohort recruitment to maximally diversify the sociodemographic profiles of participants, however, remains a contentious issue.

**Objective:**

The aim of the study was to characterize the sociodemographic profiles according to the recruitment mode of adult volunteers enrolled in a Web-based cohort.

**Methods:**

The French NutriNet-Santé Web-based cohort was launched in 2009. Recruitment is ongoing and largely relies on recurrent multimedia campaigns. One month after enrollment, participants are asked how they learned about the study (eg, general newscast or a health program on television, radio newscast, newspaper articles, Internet, personal advice, leaflet/flyers) The sociodemographic profiles of participants recruited through operative communication channels (radio, print media, Internet, advice) were compared with the profiles of those informed through television by using polytomous logistic regression.

**Results:**

Among the 88,238 participants enrolled through the end of 2011, 30,401 (34.45%), 16,751 (18.98%), and 14,309 (16.22%) learned about the study from television, Internet, and radio newscasts, respectively. Sociodemographic profiles were various, with 14,541 (16.5%) aged ≥60 years, 20,166 (22.9%) aged <30 years, 27,766 (32.1%) without postsecondary education, 15,397 (19.7%) with household income <€1200/month, and 8258 (10.6%) with household income €3700/month. Compared to employed individuals, unemployed and retired participants were less likely to be informed about the study through other sources than through television (adjusted ORs 0.56-0.83, *P*<.001). Participants reporting up to secondary education were also less likely to have learned about the study through radio newscasts, newspaper articles, Internet, and advice than through television (adjusted ORs 0.60-0.77, *P*<.001).

**Conclusions:**

Television broadcasts appear to permit the recruitment of e-cohort participants with diverse sociodemographic backgrounds, including socioeconomically disadvantaged individuals who are usually difficult to reach and retain in long-term epidemiologic studies. These findings could inform future Web-based studies regarding the development of promising targeted or general population recruitment strategies.

## Introduction

Prospective epidemiological studies are invaluable in advancing scientific knowledge; however, they require very large samples when dealing with rare outcomes or when aiming to accurately establish small-scale associations [1]. In addition, traditional large population-based studies require material logistic and operational resources for survey printing and mailing, recruitment and training of interviewers, data entry and cleaning, and follow-up management. In addition, in such volunteer-based studies, certain subgroups of the population (eg, the socioeconomically disadvantaged, the elderly, and rural area residents) are often underrepresented which restricts the range of available exposure and confounder measures and may limit the internal validity of the findings.

The inherent financial burden and the steadily declining participation rates in telephone- or mail-based data collection surveys [2] argue for the need for innovative and attractive recruitment and data collection strategies for epidemiological studies.

In recent decades, the expansion of the Internet for personal and professional use has underscored important changes in the field of mass communication and has presented unique opportunities to enroll and follow individuals while collecting a wide range of epidemiological data [3]. Indeed, these innovative technologies have rapidly evidenced valuable advantages for epidemiologic research, including substantial savings in logistic and financial resources, greater convenience to the participants regarding the time/place of survey completion, and potentially superior data quality in specific domains [4]. Use of the Internet in epidemiology is now commonly referred to as *e-epidemiology* and represents the science of epidemiological assessment using Internet-enabled digital media (eg, personal computers, tablets, and smartphones) [3]. Also, e-epidemiology has the potential of lowering social desirability effects because of guaranteed greater levels of privacy and anonymity compared with traditional in-person data collection strategies, thus limiting potential prevarication bias [5]. Over the past decade, the Internet has been used to implement intervention trials for smoking cessation, physical activity promotion, alcohol abstinence, healthy eating promotion [6-8], and medication adherence [9]. In addition, these novel technologies have been advanced as an alternative follow-up method in preexisting cohorts, such as the Black Women’s Health Study [10] and the Millennium Cohort Study [11]. However, few prospective cohort studies have used such innovative methods as the primary medium of contact, recruitment, and follow-up [12-15], and none has provided information about the profiles of enrollees according to the information source used.

Mass media campaigns, which are complementary to other outlets for the dissemination of public health messages, have shown promising results [16-18] and may be a feasible option for the recruitment of volunteers. In addition, the employment of multiple communication channels may help diversify participant backgrounds.

The NutriNet-Santé Study is a Web-based prospective cohort study launched in France in May 2009. With almost 40 million Web users older than 11 years (71% of the population), France provides an excellent context for Web-based scientific studies [19]. Unlike traditional epidemiological studies that often rely on recruitment through targeted postal mailings, telephone calls, or hospital/health insurance rosters, the NutriNet-Santé Study relies on a wide range of free-of-charge communication channels for disseminating the call for volunteers.

The aim of the present study was to evaluate the degree to which the sociodemographic profiles of the participants enrolled in the NutriNet-Santé Study on a voluntary basis varied across the communication source used. In particular, we expected that television, as a wide-reaching medium (99% of French households possess a television set), may aid in recruiting typically understudied population subgroups. Henceforth, we use the term *recruitment* in reference to the information channel by which the participants learned about the study before deciding to enroll in it.

## Methods

### Population

The present analysis was performed on data from participants in the NutriNet-Santé Study enrolled from May 10, 2009 through December 31, 2011. The rationale, design, and implementation of the study has been reported elsewhere [20]. Briefly, the NutriNet-Santé Study is an ongoing, large, Web-based prospective cohort study launched in France in May 2009. Its primary aim is to investigate the associations between nutritional factors and health outcomes and to elucidate the role of various determinants (eg, demographic, socioeconomic, cultural, and cognitive) of dietary patterns and nutritional status.

In the NutriNet-Santé Study, adult participants aged 18 years and older are recruited from the general population on a voluntary basis. All data are collected through a dedicated website [21] via adapted questionnaires using a secure and user-friendly HyperText Markup Language (HTML) interface. Individuals who fill out all baseline questionnaires (pertaining to sociodemographics, dietary behaviors, physical activity, anthropometrics, lifestyle, and health status) are included in the cohort [20]. All baseline questionnaires were first pilot-tested and compared with the traditional modalities (paper versions or dietitian interviews) [22-24]. Health events are monitored via questionnaires about hospitalizations and medication use as well as via a linkage with the national vital statistics database. The study was approved by the Institutional Review Board (IRB) of the French Institute for Health and Medical Research (IRB Inserm no: 0000388FWA00005831) and the Comité National Informatique et Liberté (CNIL no: 908450 and 909216).

Recruitment is scheduled for 5 years with an additional follow-up planned at 10 years. The call for volunteers is based on a vast, biannual, multimedia campaign (including print media, Internet websites and networks, and television and radio broadcasts).

At the launch of the study, the first campaign contained general information about the study and was carried out under the auspices of the Health Minister. Subsequent campaigns have been built around specific findings from the study (eg, calls for reduction in salt consumption, increase in fiber intake, adherence to nutritional guidelines). Before each campaign, a press conference is organized and a press release is widely disseminated within a 72-hour period. Aspects related to academic endeavor, public health interest, scientific progress, confidentiality, and convenience regarding participation (ie, ≤20 min each month) are emphasized.

Additional dissemination strategies have also been used, such as short message service (SMS) text messaging for the initial mass media campaign especially (n=400,000 recipients), leaflets/flyers (n=50,000 recipients), and a nonpaid advertising display. For example, the SMS text messages were sent by a French mobile service provider to a random sample of their customers. Nonpaid advertising displays included postings in doctor’s offices, health centers, worksites, public transportation, and on billboards, as well as video clips shown in post offices.

### Data Collection

The baseline set of questionnaires includes information about date of birth, sex, area of residence, education, employment status, household composition, occupational category, and income. At 1-month follow-up, participants are asked to provide information about how they heard about the NutriNet-Santé Study and are given 18 response options including “I don’t remember” or “other.” Each participant is allowed to select only 1 response to this question. For the present analyses, these responses have been grouped into the following 9 recruitment sources: (1) general newscast or a health program on television, (2) radio newscast, (3) newspaper article, (4) Internet website or network, (5) SMS text message sent by a service provider, (6) advice from a friend/family member/health care provider, (7) leaflet/flyer, (8) nonpaid advertising display, and (9) other (including “I don’t remember”).

### Statistical Analysis

For the present analysis, we focused on participants included in the NutriNet-Santé Study between May 10, 2009 and December 31, 2011. From the 104,020 participants meeting that criterion, we selected 88,238 individuals who provided information at 1-month follow-up on how they had learned about the study.

Income per household unit was first calculated and then divided by the number of household members (eg, 1 unit for the first adult in the household, 0.5 units for the other persons aged 14 years or older, and 0.3 units for children under 14 years of age) [25]. The occupation category for retired and unemployed people was defined as the last job held.

Sociodemographic characteristics of the sample, including age (<30 years, 30-45 years, 45-60 years, ≥60 years), education (primary/secondary, postsecondary <bachelor’s degree, ≥bachelor’s degree), current employment status (employed, unemployed, homemaker, retired, student), occupational category (never employed, farmer, manual worker, employees/medium-skilled labor, intermediate professions/skilled labor, self-employed, managerial/professional staff), type of area of residence (rural, urban <200,000 inhabitants, urban ≥200,000 inhabitants), and monthly household income (<€1200, €1200-€2700, €2700-€3700, >€3700 per household unit), are presented in a frequency/percent format for the entire sample and by recruitment source.

To better understand the selected sample, we compared the characteristics of included and excluded NutriNet-Santé Study participants using chi-square tests and Student *t* tests, as appropriate.

Crude and adjusted associations between the sociodemographic characteristics and the recruitment source were estimated using polytomous logistic regression (reference=television). Polytomous logistic regression generalizes the binary logistic regression model, allowing the dependent (outcome) variable to have more than 2 categories [26]. The multivariate model included the following covariates: age, sex, education, employment status, occupational category, area of residence, and monthly household income. Additionally, the model was adjusted for the interval between the most recent press release and the completion of the first follow-up questionnaire that included information about the recruitment source. Odds ratios (OR) and 95% confidence intervals (CI) are reported. We also performed a sensitivity analysis after exclusion of participants for which the most recent press release occurred between baseline and the first follow-up questionnaire. Tests of statistical significance were 2-sided and the type I error was set at 5%. Statistical analyses were performed using SAS software (version 9.2, SAS Institute Inc, Cary, NC, USA).

## Results

Compared to included participants, those participants excluded because of missing data from the first follow-up questionnaire were younger (39.64 years vs 43.01 years), less often retired (11.35% vs 17.62%, *P*<.001), more often students (11.37% vs 7.69%, *P*<.001), and they were more likely to report a low (<€1200) monthly income (27.90% vs 19.70%, *P*<.001).

The sociodemographic characteristics of the sample and recruitment sources and the corresponding national estimates are presented in Table 1. In all, 22.40% (19,764/88,238) of the participants were men; the mean age was 43.0 years (SD 14.5). Approximately two-thirds (58,776/86542, 67.92%) of the sample had postsecondary education; 31.64% (22,464/70,995) were employed in managerial/executive or professional positions, and 32.20% (22,857/70,995) were employees engaged in medium-skilled work. The sample included 7.80% currently unemployed individuals (6751/86,542) and approximately one-fifth of the sample reported monthly income <€1200 euros per household unit (19.69%, 15,397/78,171). Over one-fifth of the participants lived in rural areas (21.24%, 18,741/88,238). The use of various dissemination channels allowed the recruitment of a wide range of sociodemographic profiles with a sizeable portion of older people aged 60 years or older (16.48%, 14,541/88,238), individuals with low levels of formal education without postsecondary education (32.08%, 27,766/86,542), and with low income defined as <€1200/month (19.69%, 15,397/78,171).

Television was the most common source of information about the study; 34.47% (30414/88,238) of the participants reported learning about it through a general newscast or a health-related broadcast on television. Meanwhile, very few participants learned about the study from a noncommercial advertising display (0.61%, 542/88,238), SMS text messaging (0.86%, 760/88,238), or leaflets (1.69%, 1,494/88,238).

**Figure 1:**
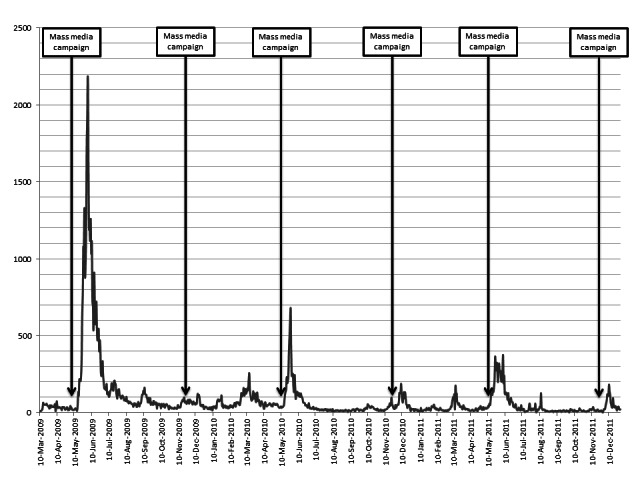
Participant enrollment per month in the NutriNet-Santé Study from March 2009 to December 2011 in relation to mass media campaigns (N=88,238).

The number of participants enrolling over time is presented in Figure 1. There were recruitment peaks following each mass media campaign. The actual number of inclusions after each media campaign varied greatly according to the key scientific message of the campaign, the breaking news at the time, and the scope of the relay by the media. The highest peak was achieved after the first multimedia campaign that introduced the study to the public and was widely disseminated.

Crude and adjusted associations between participants’ characteristics and the principal recruitment source are presented in Tables 2 and 3. In the multivariate models, compared to participants aged 30 to 45 years, older individuals (≥60 years) were more likely to be informed about the cohort through channels other than television (adjusted ORs ranging from 1.40 for radio newscast to 2.26 for personal advice). Compared to participants with postsecondary education, those with only primary/secondary education were less likely to be recruited through channels other than television (adjusted ORs ranging from 0.60 for newspaper articles to 0.77 for personal advice). Compared to employed individuals, participants who were unemployed or retired were most likely to be informed about the study through television (adjusted ORs ranging from 0.56 for newspaper articles to 0.71 for personal advice, and from 0.57 for Internet to 0.94 for newspaper articles). Compared to participants with monthly household unit income ranging from €1200 to €2299, those with low income (<€1200/month) were less likely to be informed through channels other than television (crude ORs ranging from 0.62 for radio to 0.80 for advice from a relative), but the differences did not remain significant in the multivariate model except for radio newscasts (adjusted OR 0.87, 95% CI 0.81-0.93, global *P* value for the income variable <.001).

**Table 1:** Sociodemographic characteristics of the NutriNet-Santé Study sample (N=88,238) in comparison to the general population of France [30,31].

Sociodemographic characteristic		NutriNet-Santé, n (%)	France, %
**Sex**			
	Male	19,764 (22.40)	47.6
	Female	68,474 (77,60)	52.4
**Age categories (years)**			
	<30	20,166 (22.85)	22.8
	30-44	27,372 (31.02)	24.8
	45-59	26,159 (29.65)	24.8
	≥60	14,541 (16.48)	27.6
**Education among people older than 20 years (n=86,542)**			
	Primary/secondary	27,766 (32.08)	75.3
	Some college, associate degree	26,324 (30.2)	11.9
	≥Bachelor’s degree	32,452 (37.50)	12.6
**Occupational category among nonretired people older than 20 years (n=70,995)** ^a^			
	Never employed	3401 (4.79)	18.7
	Farmers	272 (0.38)	1.1
	Manual workers	2118 (2.98)	14.7
	Employees/medium-skilled labor	22,857 (32.20)	18.1
	Intermediate professions/skilled office work	17,966 (25.31)	14.9
	Self-employed	1917 (2.70)	3.6
	Managerial/professional positions	22,464 (31.64)	9.3
**Current employment status (n=86,542)** ^b^			
	Employed	55,168 (63.75)	55.1
	Homemakers	3896 (4.50)	4.6
	Unemployed	6751 (7.80)	11.3
	Retired	15,547 (17.96)	28.7
	Students	5180 (5.99)	2.5
**Monthly income (€ per household unit)** ^c^ **(n=78171)**			
	< 1200	15,397 (19.69)	
	1200-2299	34,764 (44.47)	
	2300-3699	19,752 (25.27)	
	> 3700	8258 (10.56)	
**Area of residence**			
	Rural	18,741 (21.24)	
	Urban (population <200,000)	29,619 (33.57)	
	Urban (population >200,000)	39,878 (45.19)	
**Recruitment source**			
	Television (general newscast or a health program)	30,414 (34.47)	
	Radio newscast	14,309 (16.22)	
	Newspaper articles	9613 (10.89)	
	Nonpaid advertising display	542 (0.61)	
	Internet websites	16,807 (19.05)	
	SMS text message sent by a service provider	760 (0.86)	
	Leaflet/flyers	1494 (1.69)	
	Advice from a friend/family member/health care provider	10,172 (11.53)	
	Other	4127 (4.68)	

^a^Occupational category for unemployed people was defined as the most recent type of job held. For comparison purposes, occupational categories are shown for people who are not retired and are older than 20 years.

^b^For comparison purposes, employment status is shown for people older than 20 years.

**Table 2:** Crude associations between sociodemographic profiles and recruitment source using television as reference (n=72,264).

Sociodemographic characteristic		Recruitment source, OR (95% CI)^a,b^			
		Advice from a friend/family member/health care provider (n=10,172)	Internet websites and networks n=(16,807)	Newspaper articles (n=9613)	Radio newscast (n=14,309)
**Sex**					
	Men	1.31 (1.24-1.39)	1.19 (1.13-1.24)	1.26 (1.19-1.34)	1.46 (1.39-1.53)
	Women	1 (ref)	1 (ref)	1 (ref)	1 (ref)
**Age categories (years)**					
	<30	1.44 (1.35-1.53)	0.93 (0.88-0.98)	0.85 (0.79-0.91)	0.54 (0.51-0.58)
	30-44	1 (ref)	1 (ref)	1 (ref)	1 (ref)
	45-59	1.19 (1.11-1.27)	1.19 (1.13-1.25)	1.27 (1.20-1.35)	1.13 (1.07-1.19)
	≥60	1.86 (1.73-2.00)	1.18 (1.11-1.25)	1.55 (1.44-1.66)	0.90 (0.84-0.96)
**Education**					
	Primary/secondary	0.71 (0.67-0.76)	0.69 (0.65-0.72)	0.67 (0.64-0.72)	0.54 (0.52-0.57)
	Some college, associate degree	1 (ref)	1 (ref)	1 (ref)	1 (ref)
	≥Bachelor’s degree	1.88 (1.76-2.00)	1.72 (1.63-1.81)	1.69 (1.59-1.80)	1.81 (1.72-1.91)
**Occupational category** ^c^					
	Never employed	2.24 (2.00-2.50)	1.30 (1.17-1.45)	1.09 (0.95-1.26)	0.99 (0.87-1.13)
	Farmers	1.27 (0.85-1.89)	0.69 (0.47-1.03)	1.00 (0.65-1.55)	2.12 (1.57-2.86)
	Manual workers	0.70 (0.59-0.83)	0.77 (0.68-0.87)	0.69 (0.58-0.82)	0.87 (0.75-1.00)
	Employees/medium-skilled labor	1 (ref)	1 (ref)	1 (ref)	1 (ref)
	Intermediate professions/skilled office work	1.79 (1.68-1.91)	1.65 (1.56-1.74)	1.75 (1.64-1.87)	2.11 (1.99-2.24)
	Self-employed	1.11 (0.95-1.30)	0.96 (0.85-1.10)	0.98 (0.83-1.15)	1.67 (1.48-1.89)
	Managerial/professional positions	2.78 (2.61-2.96)	2.49 (2.36-2.62)	2.65 (2.49-2.82)	3.43 (3.25-3.63)
**Current employment status**					
	Employed	1 (ref)	1 (ref)	1 (ref)	1 (ref)
	Homemakers	0.47 (0.41-0.54)	0.43 (0.38-0.48)	0.54 (0.47-0.61)	0.63 (0.57-0.70)
	Unemployed	0.58 (0.53-0.64)	0.60 (0.56-0.65)	0.46 (0.41-0.51)	0.46 (0.42-0.50)
	Retired	1.30 (1.22-1.38)	0.86 (0.81-0.91)	1.23 (1.16-1.31)	0.78 (0.73-0.82)
	Students	1.46 (1.34-1.60)	0.78 (0.72-0.85)	0.65 (0.58-0.73)	0.46 (0.41-0.51)
**Income (€ /month per household unit)**					
	<1200	0.80 (0.75-0.86)	0.79 (0.75-0.83)	0.67 (0.63-0.72)	0.62 (0.58-0.65)
	1200-2,299	1 (ref)	1 (ref)	1 (ref)	1 (ref)
	2300-3699	1.55 (1.46-1.65)	1.37 (1.30-1.44)	1.58 (1.49-1.68)	1.60 (1.52-1.69)
	>3700	2.05 (1.89-2.22)	1.68 (1.56-1.80)	1.85 (1.70-2.01)	2.03 (1.89-2.18)
**Area of residence**					
	Rural	0.94 (0.88-1.01)	1.00 (0.94-1.06)	0.90 (0.84-0.96)	1.11 (1.05-1.18)
	Urban (population <200,000)	1 (ref)	1 (ref)	1 (ref)	1 (ref)
	Urban (population >200,000)	1.53 (1.45-1.62)	1.43 (1.37-1.50)	1.27 (1.20-1.34)	1.22 (1.16-1.28)

^a^Reference=television; for example, compared to participants aged 30-45 years, participants <30 years were less often informed about the study through the radio than through television (OR 0.54).

^b^All *P* values for the association between each sociodemographic variable and recruitment source (*P*<.001).

^c^Occupational category for retired and unemployed people defined as the most recent type of job held.

**Table 3:** Multivariate associations between the participant sociodemographic profiles and recruitment source using television as reference (n=72,264).

Sociodemographic characteristic		Reference source, adjusted OR^a^ (95% CI)^b,c^			
		Advice from a friend/family member/health care provider (n=10,172)	Internet websites and networks (n=16,807)	Newspaper articles (n=9613)	Radio newscast (n=14,309)
**Sex**					
	Men	1.10 (1.03-1.17)	1.04 (0.99-1.10)	1.05 (0.99-1.12)	1.25 (1.19-1.32)
	Women	1 (ref)	1 (ref)	1 (ref)	1 (ref)
**Age categories (years)**					
	<30	1.31 (1.22-1.41)	0.94 (0.88-1.00)	0.89 (0.82-0.96)	0.60 (0.56-0.64)
	30-44	1 (ref)	1 (ref)	1 (ref)	1 (ref)
	45-59	1.39 (1.30-1.49)	1.46 (1.38-1.54)	1.49 (1.39-1.59)	1.41 (1.34-1.50)
	≥60	2.26 (2.00-2.57)	2.07 (1.86-2.30)	1.73 (1.53-1.96)	1.40 (1.25-1.57)
**Education**					
	Primary/secondary	0.77 (0.72-0.82)	0.71 (0.66-0.75)	0.60 (0.57-0.64)	0.75 (0.71-0.80)
	Some college, associate degree	1 (ref)	1 (ref)	1 (ref)	1 (ref)
	≥Bachelor’s degree	1.54 (1.44-1.65)	1.48 (1.38-1.58)	1.53 (1.44-1.62)	1.47 (1.38-1.56)
**Occupational category** ^d^					
	Never employed	1.33 (1.15-1.54)	1.31 (1.14-1.50)	1.25 (1.04-1.50)	1.26 (1.06-1.49)
	Farmers	1.25 (0.83-1.87)	0.60 (0.40-0.89)	0.87 (0.56-1.36)	1.62 (1.19-2.20)
	Manual workers	0.80 (0.67-0.95)	0.88 (0.77-1.01)	0.84 (0.70-1.00)	1.01 (0.88-1.17)
	Employees/medium-skilled labor	1 (ref)	1 (ref)	1 (ref)	1 (ref)
	Intermediate professions/skilled office work	1.43 (1.33-1.54)	1.35 (1.28-1.44)	1.30 (1.21-1.39)	1.50 (1.41-1.60)
	Self-employed	0.97 (0.82-1.13)	0.84 (0.74-0.96)	0.80 (0.68-0.94)	1.31 (1.15-1.49)
	Managerial/professional positions	1.58 (1.46-1.71)	1.58 (1.48-1.69)	1.46 (1.35-1.59)	1.73 (1.62-1.86)
**Current employment status**					
	Employed	1 (ref)	1 (ref)	1 (ref)	1 (ref)
	Homemakers	0.60 (0.52-0.70)	0.50 (0.45-0.56)	0.66 (0.57-0.75)	0.84 (0.76-0.94)
	Unemployed	0.71 (0.65-0.79)	0.69 (0.64-0.75)	0.56 (0.50-0.62)	0.60 (0.55-0.66)
	Retired	0.83 (0.74-0.93)	0.57 (0.51-0.63)	0.94 (0.84-1.05)	0.63 (0.57-0.70)
	Students	1.64 (1.44-1.88)	1.02 (0.91-1.16)	0.98 (0.83-1.15)	1.09 (0.94-1.27)
**Income (€ /month per household unit)**					
	<1200	0.94 (0.87-1.01)	1.04 (0.98-1.10)	0.94 (0.87-1.01)	0.87 (0.81-0.93)
	1200-2299	1 (ref)	1 (ref)	1 (ref)	1 (ref)
	2300-3699	1.16 (1.09-1.23)	1.02 (0.97-1.08)	1.16 (1.09-1.23)	1.15 (1.09-1.22)
	>3700	1.25 (1.14-1.37)	1.00 (0.92-1.08)	1.08 (0.99-1.19)	1.12 (1.03-1.21)
**Area of residence**					
	Rural	1.01 (0.94-1.09)	1.05 (0.99-1.12)	0.95 (0.88-1.02)	1.17 (1.11-1.24)
	Urban (population <200,000)	1 (ref)	1 (ref)	1 (ref)	1 (ref)
	Urban (population >200,000)	1.30 (1.22-1.37)	1.25 (1.19-1.31)	1.11 (1.05-1.18)	1.02 (0.97-1.08)

^a^For each predictor, the respective OR is adjusted for the remaining variables and for the interval (in days) between the most recent press release (days) and the survey completion.

^b^Reference=television; for example: compared to participants aged 30-45 years, participants <30 years were less often informed about the study through the radio than through television (OR 0.60).

^c^All *P* values for the association between each sociodemographic variable and recruitment source (*P*<.001).

^d^Occupational category for retired and unemployed people defined as the most recent type of job held.

## Discussion

### Principal Results

We examined how the sociodemographic profiles of participants in a large, Internet-based cohort differed according to the recruitment source used. The use of a number of different dissemination channels allowed the recruitment of a sizeable and diverse cohort. As expected, most participants enrolled after hearing about the study on television because this medium has the widest reach. In particular, television announcements permitted the recruitment of a larger proportion of members of population subgroups (eg, those belonging to lower socioeconomic strata) that are not typically well represented in population-based epidemiologic research. However, the elderly were more likely to be informed about the cohort through channels other than television suggesting that television did not necessarily represent the best information medium for all population subgroups. In turn, radio, newspapers, Internet, and personal advice also proved to be substantial means of disseminating information about this epidemiologic study to encourage participation.

Overall, the recruitment of participants from a wide range of sociodemographic backgrounds provides the study with a broader range of exposures and confounders than does a more homogeneous sample. This is of important concern when the overarching aim is to estimate associations between certain exposures and health/disease outcomes, as in the NutriNet-Santé Study [1,13,27,28].

### Comparison With Prior Work

Among the initial efforts to recruit Web-based cohorts, in examining the literature we identified 1 study calling for participation in a cohort of smokers intending to quit, by using existing Internet panels intended for market research [12]. Another study recruited nurses and midwives through targeted email distribution [14]. A birth cohort study employed hospital advertising [15] and a pregnancy planning study made recruitment announcements on a health-related website [13]. To the best of our knowledge, this is the first study pertaining to the quantitative description of participant profiles according to the recruitment source.

### Limitations

The main limitation of this analysis was the lack of information on participation/refusal rates because the call for participation was not delivered to a predefined and exhaustive list of randomly selected individuals. In turn, the results need to be interpreted in light of the fact that the actual effectiveness of the media channels cannot be estimated. Second, misclassification of recruitment source is also possible because the question about information source was asked 1 month after baseline. Additional misclassification about the recruitment source cannot be ruled out because participants may have been exposed to several information sources. For example, if a press release occurred during the interval between baseline and the completion of the first follow-up questionnaire, this may have led to an overreporting of the related information channels. However, the sensitivity analysis performed after removing these participants did not substantially modify the findings.

### Cohort Profile and Aspects Related to Future Analyses of the Nutrinet-Santé Data

Concerns have been raised regarding the selection bias potentially inherent in cohorts followed via the Internet. Web users may present particular sociodemographic profiles, especially regarding sex, age, and education [3,10,29]. Our findings suggest that vast media campaigns coupled with expanding Internet access permit reaching individuals from a wide range of sociodemographic backgrounds.

As compared with national estimates [30-32], the NutriNet-Santé Study sample included proportionally more women (77.60% vs 52%) and individuals of relatively high socioeconomic status (managerial/professional staff excluding retired people: 31.64% vs 9.3% nationally; postsecondary education: 67.92% vs 24.3% nationally). This is consistent with existing knowledge regarding the characteristics of participants in volunteer-based studies dealing with health and nutrition [1]. Any additional selection bias is likely negligible given the wide range of recruitment channels used and the widespread access to the Internet in France, with a current penetration rate of 77% [33]. For example, 27% of the Web users in France are older than 50 years, which is relatively close to the respective proportion of individuals in that age range in the NutriNet-Santé Study (36%).

As suggested in the literature [28], the response rate and the related potential lack of representativeness, as observed in our study, is not critical in an etiologic context. However, this remains a key issue when descriptive information is provided. In the present study, 1 of the most important aspects pertained to the sizeable and heterogeneous cohort, including a wide range of sociodemographically diverse profiles, thus ensuring material variability in exposure factors. The use of various recruitment channels allowed meeting this objective by including specific subgroups of the population, such as those older than 60 years, those with low income, and/or with low levels of formal education.

It has also been recently postulated that in the context of a Web-based cohort of volunteers, the generalizability of etiologic findings depends on whether the studied associations might differ between those with and without Internet access, which seems unlikely [13].

Along with being geographically unrestricted, Web-based prospective cohorts present with many logistic and cost advantages compared to traditional samples [3,4,11,22,34,35]. Furthermore, use of the Internet entails lower social desirability effects, which could also facilitate the recruitment of participants exhibiting unhealthy behaviors or those with socially undesirable/stigmatizing backgrounds.

### Conclusion

In conclusion, the present findings fill gaps in current knowledge about cohort recruitment by providing new insights about the sociodemographic profiles of adult volunteers. Our Web-based cohort study uses recurrent mass media campaigns and numerous public outlets for encouraging participation. The various information channels allow the inclusion of individuals with diverse sociodemographic profiles. As expected, television appeared to be the most promising channel for reaching potential participants representing a wide range of sociodemographic backgrounds, including subgroups that are usually difficult to involve in long-term epidemiologic studies. Our findings could inform future Web-based studies regarding the development of operative targeted or general population recruitment strategies.

## Abbreviations

HTML: HyperText Markup Language
IRB: Institutional Review Board
SMS: short message service
